# Comparative Response of Brain to Chronic Hypoxia and Hyperoxia

**DOI:** 10.3390/ijms18091914

**Published:** 2017-09-07

**Authors:** Laura Terraneo, Michele Samaja

**Affiliations:** Department of Health Science, University of Milan, I-20142 Milano, Italy; laura.terraneo@gmail.com

**Keywords:** hypoxia, hyperoxia, chronic stress, cerebral tissue, signaling pathways, oxidative stress, brain injury, oxygen sensing

## Abstract

Two antithetic terms, hypoxia and hyperoxia, i.e., insufficient and excess oxygen availability with respect to needs, are thought to trigger opposite responses in cells and tissues. This review aims at summarizing the molecular and cellular mechanisms underlying hypoxia and hyperoxia in brain and cerebral tissue, a context that may prove to be useful for characterizing not only several clinically relevant aspects, but also aspects related to the evolution of oxygen transport and use by the tissues. While the response to acute hypoxia/hyperoxia presumably recruits only a minor portion of the potentially involved cell machinery, focusing into chronic conditions, instead, enables to take into consideration a wider range of potential responses to oxygen-linked stress, spanning from metabolic to genic. We will examine how various brain subsystems, including energetic metabolism, oxygen sensing, recruitment of pro-survival pathways as protein kinase B (Akt), mitogen-activated protein kinases (MAPK), neurotrophins (BDNF), erythropoietin (Epo) and its receptors (EpoR), neuroglobin (Ngb), nitric oxide (NO), carbon monoxide (CO), deal with chronic hypoxia and hyperoxia to end-up with the final outcomes, oxidative stress and brain damage. A more complex than expected pattern results, which emphasizes the delicate balance between the severity of the stress imposed by hypoxia and hyperoxia and the recruitment of molecular and cellular defense patterns. While for certain functions the expectation that hypoxia and hyperoxia should cause opposite responses is actually met, for others it is not, and both emerge as dangerous treatments.

## 1. Introduction

Hypoxia, or insufficient supply of oxygen (O_2_) with respect to the demand, represents a relevant clinical and environment condition that afflicts millions of people worldwide and constitutes an important source of social and economic distress. The burden imposed in 2010 in the US only by chronic obstructive pulmonary disease, a single pathological situation that involves hypoxia, was projected to be approximately US$49.9 billion [1], and cerebral palsy, caused by hypoxic-ischemic encephalopathy, requires costs around US$9 million [2]. Patients suffering from hypoxia-linked conditions are often treated by inhalation of hyperoxic or hyperbaric atmospheres, thereby potentially establishing a reversed condition of excessive O_2_ supply with respect to demand, which may not be free of deleterious effects [3,4]. Besides constituting a specific clinical entity, hyperoxia may also represent a valuable experimental bench to test various hypotheses on the mechanism of action of hypoxia. In theory, O_2_ shortage and O_2_ excess should have divergent effects, but if this pattern is not observed, then it is likely that other stressors not directly linked to the lack/excess of O_2_ are involved, leading to a condition where the responses to hypoxia and hyperoxia depart from divergence and may even overlap. Thus, comparing the responses to hypoxia and hyperoxia may facilitate the identifying of areas of research that clarify the role of O_2_ in various cellular and molecular mechanisms.

The purpose of this review article is to assess the molecular and cellular responses to hypoxia and hyperoxia in cerebral tissue, a reliable experimental model for several reasons. First, most the systemic responses to stress converge into the defense of the brain function, which emphasizes that this function is one of the most protected ones in the body. For example, the observations that cerebral blood flow is not coupled to the metabolic rate for O_2_ in physiologically activated brain states [5] and that the brain possesses efficient blood flow autoregulatory potential to preserve its oxygenation at varying hematocrits [6] point to the paradigm that the evolution has placed the brain in a high-priority rank. Second, the low regenerative potential of neurons enables apoptosis to emerge as reliable marker of brain injury. Third, the high metabolic rate and the extensive recruitment of the oxidative metabolism as the major source for biologic energy production, as well as the lack of O_2_ stores, force cerebral tissue to rely entirely on continuous supply of O_2_ and on cerebral perfusion for its function [7]. Whenever the O_2_ availability is altered for any environmental or pathophysiological cause, the ensuing condition may surge as a potentially harmful challenge for brain function, against which tissue must react in order to preserve its viability. Whether the hypoxic challenge is well known to be deleterious for brain tissue from several studies performed in vitro, in vivo and in humans (reviewed later in Section 12 Brain injury), it is still controversial if hyperoxia may be considered dangerous, although the apparent trend is that hyperoxia increases apoptosis [8], decreases neuroprotection [9], and favors the pro-oxidant state [10].

In addition to the hypoxia vs. hyperoxia comparison, the concept of chronic vs. acute stress deserves further consideration. Indeed, although hypoxia represents a potentially lethal condition, most mammal tissues have a considerable reserve that enables recruitment of defense mechanisms to grant survival during acute episodes. But clearly, when sustained or chronic, hypoxia is predicted to imply greater effort to balance its harmful effects through recruitment of gene- and protein-based compensatory mechanisms. Here, we review the existing literature regarding the ability of brain to adapt to chronic hypoxia and chronic hyperoxia with analysis of the metabolic and molecular changes.

In this article, we will focus primarily in published findings gathered in brain tissue, but the lack of information may sometimes force the use of findings gathered in tissues other than brain, which will be accurately identified for the sake of clarity. We apologize for neglecting many valuable contributions in this expanding sector.

## 2. Metabolic Response

Any protective mechanism triggered by hypoxia or hyperoxia should in principle be centered in the re-establishment of the altered O_2_ supply/O_2_ demand balance. In the case of hypoxia, this goal can be exploited by decreasing the O_2_ demand, increasing the O_2_ supply, or a combination of both. Some animals have the ability to reduce the O_2_ demand through a condition called hypometabolism, but in the human brain this condition is associated with almost immediate insurgence of neurological disorders. Alternatively, the O_2_ demand can be reduced by increasing the recruitment of anaerobic mechanisms, but in the brain tissue the aerobic-to-anaerobic switch is of limited value because of its reduced glycolytic capacity [11], which impairs this kind of defense. Indeed, O_2_ shortage is always associated to early signs of failure represented by marked falls in pH and tissue creatine phosphate levels [12], followed by nearly immediate dysfunction of Na^+^/K^+^ ATPase that finally leads to lethal ion imbalance [13]. The poor brain plasticity in terms of metabolic adjustment and its inability to improve the metabolic efficiency by switching to anaerobic energy-yielding pathways [7] lead to a situation whereby the defense of the brain function against O_2_ shortage is exploited either through triggering pro-survival pathways, or through improving brain oxygenation. It should, however, be pointed out that, although the brain is thought to be an insulin-insensitive organ, several studies [14] are introducing the concept that insulin may play important roles in the central nervous system too, thereby yielding a new light to glucose-dependent responses to altered O_2_ supply/O_2_ demand balance. At present, though, it is difficult to focus on insulin as an anti-hypoxia/hyperoxia molecule.

With the exception of early observations, namely that of exposing rats to F_I_O_2_ = 1.0 [For consistency, we express the degree of hypoxia/hyperoxia as the fraction of O_2_ in the various experimental models (F_I_O_2_). This notation comprises the fraction of O_2_ in inspired air in the case of in vivo studies, the O_2_ content in the atmosphere where cells are cultured in the case of in vitro studies, etc. F_I_O_2_ values <0.21 and >0.21 refer to hypoxia and hyperoxia, respectively] under hyperbaric conditions (2.5 atm) reduces the blood glucose level in rats [15], only a few studies have examined the effects of hyperoxia on bioenergetics metabolism. The prevalent context of such studies is a post-stroke situation where hyperoxia is used as part the therapeutic interventions aimed at salvaging neurons at risk. For example, treatment of patients with acute brain injury by administrating gas mixtures with F_I_O_2_ = 0.6–1.0 for 2 h improves the redox state, reduces the lactate/pyruvate ratio, a surrogate measurement for mitochondrial O_2_ availability, thus highlighting that in this clinical setting the neurologic dysfunction arises as a O_2_ diffusion failure [16]. Clearly, experimental studies in post-stroke patients imply that the damage inferred by the preceding ischemic condition may considerably complicate data interpretation, and some reports indeed emphasize variable effects of hyperoxia on the tissue lactate/pyruvate ratio [17]. In a preliminary clinical trial aimed at assessing whether a conservative protocol for O_2_ supplementation could improve outcomes in patients admitted to intensive care units, the majority of whom, however, were affected by respiratory failure rather than brain injury, resulted in lower mortality than in patients receiving the conventional hyperoxia treatment [18], but further multi-center trials are needed to assess this critical issue.

**Figure 1:**
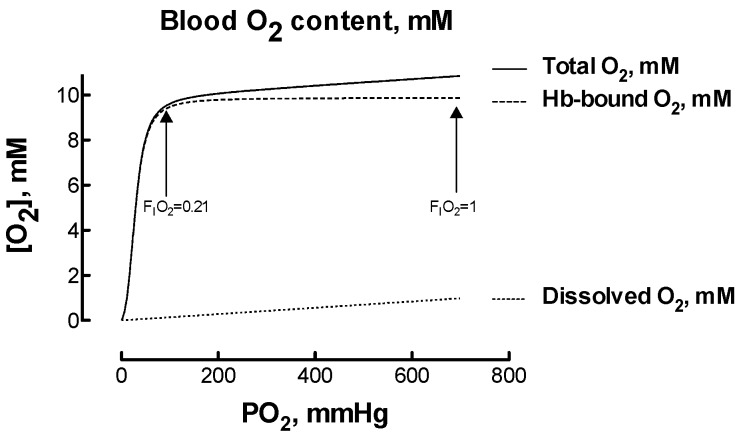
Oxygen content in arterial blood at varying PO_2_. The O_2_ content is calculated assuming a blood hemoglobin (Hb) concentration of 16 g/dL, the O_2_ solubility coefficient of 0.0014 millimoles/L/mmHg [22] and the Hb-O_2_ equilibrium curve in healthy humans [23].

Hyperoxia-induced hypocapnia, or reduced carbon dioxide in the blood, usually resulting from hyperventilation, deserves attention as it might reduce organ blood flow paradoxically inducing hypoxia [19]. This possibility raised debate. On one hand, it was hypothesized that the hypocapnia-induced decrease in cerebral blood flow may offset any increase in blood O_2_ content and reduce actual O_2_ delivery [20]. On the other hand, it was observed that the magnitude of the effects led by hypocapnia may be of little clinical relevance [4]. How to calculate the changes in blood O_2_ amounts that are involved during hypoxia and hyperoxia? When healthy subjects with arterial O_2_ saturation in the 95–100% range breath an atmosphere containing F_I_O_2_ > 0.21, the amount of O_2_ physically dissolved in blood plasma increases linearly with F_I_O_2_, yet the amount of O_2_ bound to hemoglobin (Hb) does not vary considerably because Hb is already almost fully saturated with O_2_ (Figure 1). While at PO_2_ = 100 mmHg, close to the normal value in arterial blood when breathing F_I_O_2_ = 0.21, the amount of dissolved O_2_ is roughly 1.5% of the total blood O_2_ content, this amount raises to 9.5% when breathing F_I_O_2_ = 1. Therefore, increasing F_I_O_2_ from 0.21 to 1 corresponds to a six-time increase in dissolved O_2_, to be compared to a 13% increase in the total blood O_2_ content. It remains matter of investigation if the capillary O_2_ delivery relies more on free dissolved O_2_ (i.e., the O_2_ partial pressure or PO_2_) or on total blood O_2_ content (i.e., the product [Hb] × the fractional O_2_ saturation). Clearly, for being released to tissues, O_2_ must first unbind from red cell Hb and then cross the red cell membrane. An attempt to model this process on a theoretical basis has limited validity due to the large number of assumptions needed, but the red cell membrane does not emerge as a competitive element of resistance in the favor of factors as the Bohr effect and the O_2_ unloading characteristic of Hb [21]. 

## 3. Oxygen Sensing

An essential step in the body’s response to environment and systemic changes, the sensing system triggers selected molecular and cellular mechanisms aimed at survival in response to the detected alteration. In the brain, the availability of mechanisms that rapidly detect the changes in O_2_ supply and orchestrate the response is of crucial importance to preserve viability [24]. Brief hypoxia is sensed by modulating a few reactions, primarily those catalyzed by NADPH oxidase, nitric oxide (NO) synthases (NOS) and heme oxygenase (HO):

NADPH + 2 O_2_ ↔ NADP^+^ + 2 O_2_^−^ + H^+^ (1)

2 l-arginine + 3 NAD(P)H + 3 H^+^ + 4 O_2_ ↔ 2 l-citrulline + 2 NO + 3 NAD(P)^+^ + 4 H_2_O (2)

Heme^++^ + 3 O_2_ + 4 NADPH + 4 H^+^ ↔ biliverdin + Fe^++^ + CO + 4 NADP^+^ + 3 H_2_O (3)

In these reactions, molecular O_2_ appears as one of the reactants, and at least one of the products has a second messenger function: the superoxide anion, which rapidly converts into toxic reactive O_2_ species (ROS) that also serve as messengers for a variety of functions, NO and carbon monoxide (CO), respectively. However, these quickly activated systems presumably become insufficient to grant survival for long-term or severe hypoxia, and are overruled by other more persisting transcriptional systems such as those orchestrated by the hypoxia-inducible transcription factors (HIFs). HIFs’ are probably the most important transcription factors that mediate the hypoxic signaling, because they regulate the expression of genes that mediate adaptation to low O_2_ as, for example, the genes that form part of the neuroprotective response by enabling cells to activate erythropoiesis, growth and proliferation.

HIFs are heterodimers formed by an α-subunit (HIF-α) and a β-subunit (HIF-β) [25]. Although required for the hypoxic response, the β-subunit, constitutively expressed in all cells and also known as aryl hydrocarbon receptor nuclear translocator (ARNT), is not affected by O_2_. By contrast, the expression of the α-subunit is directly regulated by the O_2_ level. Under normal O_2_ conditions corresponding to breathing gas mixtures with F_I_O_2_ = 0.21, the proline residues in the HIF molecule are hydroxylated by prolyl-hydroxylases (PHD), which request O_2_ and 2-oxoglutarate as substrates [26,27,28]:

l-proline + 2-oxoglutarate + O_2_ ↔ hydroxyproline + succinate + CO_2_ (4)

When the proline residues are hydroxylated, the HIF α-subunit interacts with the von Hippel-Lindau protein tumor suppressor protein (pVHL) forming a complex that binds ubiquitin ligase and is targeted for degradation by the proteasome [29]. By contrast, low O_2_ levels reduce the activity of PHDs with consequent low hydroxylation of the proline residues and stabilization of the HIF α-subunit, which translocates into the nucleus, forms a dimer with the β-subunit and, after recruiting transcriptional coactivators, binds the hypoxia responsive element located in the target genes [30,31]. Remarkably, though, not only are the proline residues the HIF α-subunits hydroxylated as in reaction 4, but the asparagine residues also undergo a similar reaction catalyzed by asparaginyl hydroxylase [32]:

l-asparagine + 2-oxoglutarate + O_2_ ↔ hydroxyasparagine + succinate + CO_2_ (5)

At odds with PHDs, the hydroxylation of an asparagine residue in the HIF α-subunits abrogates its interaction with the ubiquitin system, preventing transcriptional activation, and is therefore labeled as factor inhibiting HIF (FIH) [33]. Currently, three isoforms of α-subunit are known.

1. HIF-1α, discovered in liver tissue and described as a hypoxia-inducible nuclear factor able to bind the erythropoietin (Epo) gene [34]. HIF-1α regulates more than 2% of the genes in human vascular endothelial cells [35] and is today recognized as regulator of the vast majority of hypoxia-inducible genes responsible for the cell adaptation to hypoxia, including angiogenesis, anaerobic metabolism, mitochondrial biogenesis and others. The Michaelis-Menten (Km) value for O_2_ in the PHD reaction lies in the range 40–150 mmHg [36], which is above normal PO_2_ in most organs. This implies not only that HIF-1α becomes activated even during normoxia, but also that HIF-1α becomes stabilized almost linearly in a wide F_I_O_2_ range that includes moderate hyperoxia. Thus, breathing normoxic (F_I_O_2_ = 0.21) atmospheres may induce some response in terms of HIF-1α, which in brain is expressed principally in neurons, astrocytes, ependymal cells and endothelial cells [37]. Chronic hypoxia (F_I_O_2_ = 0.1 for 15 days) has the capacity to overexpress brain HIF-1α by at least 10 times with respect to normoxia, comparable to the overexpression observed in the muscle and kidney cortex, and much higher than that observed in the heart, liver and kidney medulla [38]. Later, we will review how hyperoxia has positive counterintuitive effects on the accumulation of HIF-1α.
2. HIF-2α, also known as EPAS-1 (endothelial PAS domain protein 1), is principally expressed in endothelial cells [39], including brain capillary endothelial cells [40] and, under hypoxia induction, also in brain, heart, lung, kidney, liver, pancreas, and intestine [41]. HIF-2α, which shares 48% sequence homology with HIF-1α [39], regulates less genes than HIF-1α, yet playing a more important role in liver [42] and renal [43] erythropoiesis. While HIF-1α appears to be the isoform more expressed under short periods of intense hypoxia, HIF-2α is active during prolonged mild hypoxia [44]. In support of this view, the ability of the Tibetan population to adapt to high altitude is related to a mutation of the gene encoding HIF-2α [45]. In addition, the expression of Epo, a neuroprotective substance discussed below, is more dependent on HIF-2α than HIF-1α [46,47].
3. HIF-3α [48] is expressed in the cerebellum Purkinje cells and in the eye corneal epithelium [49]. Its role, less clear than that of HIF-1α and HIF-2α, appears to linked to that of the inhibitory PAS protein [50].

Despite their name, and the relationship with the Km of PHD outlined above, HIF’s accumulate not only during hypoxia, but also during hyperoxia. In rats exposed to F_I_O_2_ = 0.5 for 3 weeks, both HIF-1α and HIF-2α accumulate in the brain during the first week of exposure, followed by a progressive decline [51]. In mice exposed to F_I_O_2_ = 0.3 for 28 days, neurons display marked increase of nuclear HIF-2α, unrelated to oxidative stress [9]. These findings in brain tissue are indirectly confirmed in other organs and tissues as growing prostate cancer [52], newborn rats hepatocytes and liver hemopoietic cells [53] as well as myocardium [54]. The reasons for the HIF’s sensitivity to hyperoxia are not yet clear. The observation that the increase in HIF proteins is not related with HIF mRNA, but rather to the decreased expression of PHD-2, the major isoform that controls the hydroxylation of HIF-1α and HIF-2α, indicates that HIF’s overexpression during hyperoxia is not linked with increased protein synthesis but rather to diminished degradation [51]. As the Km value for O_2_ in the PHD reaction is in the 40–150 mmHg range [36], a direct proportionality is expected between O_2_ supply and the activity of PHDs, which translates into faster HIF’s degradation. Yet, the apparent rate of HIF targeting to the ubiquitination pathways may not be the central feature for HIF accumulation in the cell. Many reports show that the increase in HIF may be associated with ROS production at complex III of the mitochondrial respiratory chain, that inhibits PHD-2 [55,56]. However, it was also observed that long-term breathing F_I_O_2_ = 0.3 causes less oxidative stress than breathing F_I_O_2_ = 0.1, yet the level of HIF-2α is similar in the two situations [9]. Therefore, more work is needed to elucidate this critical aspect, and the recent finding of a molecular mechanism for HIF-1α activation that is alternative to hydroxylation and rather depends on the cyclic adenosine mono phosphate-protein kinase A (cAMP-PKA) pathway, could provide an explanation for this apparently contradictory behavior [57].

The hyperoxia-dependent increase of the HIF machinery was indirectly confirmed in experiments performed in hemorrhaged rats that were exchange-transfused with a solution containing Hb molecules engineered in order to favor persistence in the vascular bed [58] and reduced reactivity toward NO [59]. The increase in the blood O_2_ capacity by 25–30% with respect to the control condition (hemorrhaged rats transfused with a non-O_2_ carrying solution), doubled both the brain level of HIF-1α and the number of neurons over-expressing HIF-1α in a setting where Hb-related toxicity was reduced to a minimum [58]. Interestingly, the use of an Hb solution with higher oxidant power nearly blunted the number of neurons over-expressing HIF-1α, indicating that the main controller of HIF-1α expression in hyperoxia may not be simply referred to a post-translational hypoxia-driven effect, but rather to a complex interaction of various players that is to be worked out. 

## 4. Protein Kinase B

The PI3K-Akt signaling pathway plays a pivotal role to promote survival in a wide range of neuronal cell types [60]. The signaling driven by Akt, also known as protein kinase B, a serine/threonine kinase [61,62], is involved in a variety of neuronal protective mechanisms, including those mediated by the nerve growth factor [63], the insulin-like growth factor I [64], adenosine [65] and renin inhibitors [66]. The PI3K-Akt pathway is activated in human primary astrocytes cultured at F_I_O_2_ = 0.01 for 24 h to increase both the expression level of Akt and its phosphorylation [67]. In the in vivo brain, chronic hypoxia (F_I_O_2_ = 0.1 for 28 days) represents a stressor able to induce phosphorylation, hence activation, of Akt [9]. 

The main mechanism by which Akt prevents cell death is through the preservation of the mitochondrial membrane integrity and the prevention of cytochrome c release from mitochondria [68]. Hypoxia-induced neuroprotection in cerebellar neurons cultured at F_I_O_2_ = 0.05 for 9 h indeed requires Akt phosphorylation for its exploitation [69]. In human cerebral endothelial cells, a more complex mechanism occurs that involves the phosphorylation and activation of the anti-apoptotic factor survivin, via a PI3K/Akt-dependent process that reduces the cleavage of PARP-1, a substrate of caspase, and hence attenuates caspase activation [70]. Akt phosphorylation is also involved in the mechanism underlying the protection against anoxic neuronal death exploited by nicotinamide, which activates the Akt-dependent phosphorylation of Bad, thereby preventing the increase in mitochondrial permeability and the subsequent cytochrome c release [71]. 

A few investigations have shown that hyperoxia is associated with the reversed effect, i.e., decreases Akt expression and/or phosphorylation. For example, in rat pups breathing F_I_O_2_ = 0.4–0.8, p-Akt decreases progressively with time until t = 12 h, followed by a partial return to baseline value in the following 12 h [72]. The decrease in p-Akt was also observed in mice brains upon exposure to F_I_O_2_ = 0.3 for 28 days [9]. As a whole, these observations suggest that the p-Akt signaling is O_2_-dependent, i.e., it increases in hypoxia while it decreases in hyperoxia. 

## 5. Mitogen-Activated Protein Kinases

In addition to the PI3K-Akt signaling pathway, another crucial survival pathway involved in neuroprotection against a variety of stressors such as hypoxia, anoxia and the oxidative stress, is the mitogen-activated protein kinases (MAPKs), a family of serine/threonine kinases that comprises the extracellular signal-regulated kinases (ERK1/2), associated with survival as shown in suprachiasmatic nucleus cells challenged with excess glutamate [73], and the stress-activated protein kinases JNK and p38, implicated in cell death [74]. In cortical neuron cells challenged with anoxia for 24 h, the ERK signaling and its upstream MAPK/extracellular signal-regulated kinase (MEK) confers protection via phosphorylation/inactivation of Bad, a member of the pro-apoptotic mechanism [75]. The MAPK pathway is also required to activate the brain-derived neurotrophic factor (BDNF) [76], which displays relevant neuroprotective functions (see below). With the exception of a study in rat pups breathing F_I_O_2_ = 0.4–0.8, where p-ERK1/2 decreases with time until t = 12 h followed by full recovery in the following 12 h [72], no other studies, to our knowledge, have examined the effects of hyperoxia on the MAPK signaling pathways. In a model constituted by lung epithelial cells, it was found that F_I_O_2_ = 0.95 upregulates JNK and p38 thereby inducing oncosis rather than apoptosis, two processes with similar upstream but divergent downstream events that may finally lead to hyperoxia-induced inflammatory lung injury [77]. Furthermore, F_I_O_2_ = 0.95 increases JNK and ERK1/2 activation in mice lungs contributing to ventilator-induced lung injury, indicating that hyperoxia favors apoptotic cell death through activation of the JNK and ERK1/2 pathways [78].

## 6. Brain-Derived Neurotrophic Factor

Neurotrophins, a family of growth factors essential for neuron development, differentiation, maturation and survival, have the ability to rescue cerebellar granule neurons from oxidative stress-mediated apoptotic death through recruitment of the PI3K-Akt and the MAPK pathways [79]. Among neurotrophins, the brain-derived neurotrophic factor (BDNF) plays an important role in neurogenesis and synaptic transmission [80] by binding to TrkB and p75 receptors, both expressed in brain [81]. BDNF protects cultured cortical neurons against hypoxia via activation of the ERK1/2 and PI3K-Akt, but not the p38 pathways [82]. Negatively affected by pathological cognitive decline, BDNF has been proposed as a molecular marker of neuroplasticity [83]. In addition, BDNF, together with the calcium-binding protein B (S100B), is linked to the psychological and cognitive impairment common in long-term (1–5 years) migrators to 4500 m altitude (F_I_O_2_ = 0.12 approximately) [84], despite possible partial adaptation to altitude. 

By contrast with hypoxia, acute (6 h) hyperoxia in the infant rat brain markedly down-regulates BDNF, as well as neurotrophins 3 and 4, followed by near complete recovery in the subsequent 15–20 h, thereby contributing to hyperoxia-linked apoptotic neurodegeneration [72]. A similar outcome was observed in the carotid body of neonatal rats treated with F_I_O_2_ = 0.6 from 24–36 h prior to birth until 3 day of age: the BDNF protein, but not its mRNA level, reduced by 70%, potentially contributing to carotid body hypoplasia [85]. The reported observations thus suggest that BDNF is O_2_-dependent similarly to Akt and MAPK, i.e., it increases in hypoxia while decreasing in hyperoxia.

## 7. Erythropoietin

Erythropoietin (Epo), commonly known as a pleiotropic cytokine produced in the kidney [86], is responsible for erythropoiesis through interaction with the Epo receptor (EpoR) of erythroid progenitors [87]. Epo is also produced in brain astrocytes and neurons [88] and EpoR is expressed in the brain [89]. Epo-EpoR binding, localized in several areas of the brain [90], was found to be upregulated under hypoxic conditions [91]. Indeed, HIF-1α binds to the Epo enhancer sequence in response to reduced O_2_ supply [92], suggesting the contribution of the Epo-EpoR system to neuroprotection against hypoxia [93], ischemia [94] and hypoxia/reoxygenation injury [95]. The mechanism underlying Epo-induced neuroprotection involves the prevention of DNA fragmentation through direct modulation of Akt phosphorylation, which results into reduced mitochondrial membrane depolarization and reduced cytochrome c release [96]. Up-regulation of the Epo-EpoR system in human ischemic/hypoxic brains further suggests a key role of this system for endogenous neuroprotection [97], as confirmed by independent studies performed in primary cultures of rat hippocampal neurons, which also show a link with the BDNF response [98], as well as in a model of carotid clamping where the Epo increase in the jugular vein blood is viewed as an endogenous protection mechanism aimed at limiting the ischemia/reperfusion damage [99]. Epo therapy is presently under consideration to improve neuroprotection against hypoxia/ischemia especially in pre-term babies [100]. In a mice pup model (F_I_O_2_ = 0.1), treatment with human recombinant Epo rescues hypoxia-induced decrease in hippocampal neurogenesis and oligodendrocyte progenitor populations, thereby providing clues for Epo-based therapy in pediatric chronic hypoxia [101]. Furthermore, Epo therapy markedly improves neurologic outcomes in newborns affected with hypoxic-ischemic encephalopathy [102]. To prevent excessive erythropoiesis, an Epo derivative devoid of erythropoietic activity, e.g., carbamylated Epo, was found to represent a pharmacological tool to protect brain in mice exposed to F_I_O_2_ = 0.1 for 15 days [103]. Interestingly, Epo is able to increase NO production by human umbilical vein endothelial cells (HUVEC) cultured at F_I_O_2_ = 0.02 through activation of eNOS thereby potentially improving vascular NO availability [104]. 

The effect of hyperoxia on brain Epo production was examined in two studies with controversial outcomes. While F_I_O_2_ = 0.5 for 3 weeks up-regulates Epo expression in mouse brain consequently to increased HIF-2α [51], F_I_O_2_ = 0.3 for 4 week is unable to elevate Epo expression, but nevertheless it increases the expression of EpoR [9]. As the Epo-EpoR system requires the simultaneous presence of both Epo and EpoR for exploiting neuroprotection, it remains matter of investigation whether the recruitment of this system depends directly on O_2_ availability or other components may eventually come into play.

## 8. Neuroglobin

A vertebrate globin with high affinity for O_2_ [105], namely neuroglobin (Ngb), is expressed in the neurons of the cerebral cortical regions, as well as in subcortical structures such as thalamus and hypothalamus, nuclei of cranial nerves in the brainstem and cerebellum [106]. Induced by hypoxia and ischemia, Ngb is believed to protect neurons from hypoxic and ischemic cell death. Inhibiting its expression in cortical neuron cultures with an antisense oligodeoxynucleotide reduces neuronal survival, whereas its overexpression provides protection against hypoxia [107]. However, as a matter of facts, although Ngb mRNA expression is significantly enhanced in cultured neuronal cell lines cultured at F_I_O_2_ = 0.01 or less for 24 h, its expression failed to increase in in vivo rat brains after transient global ischemia [108]. Likewise, although Ngb expression occurs in focal regions of the brain of the normoxic adult mouse, no significant changes are observed in response to F_I_O_2_ = 0.1 for 2 weeks [109]. It was suggested [108] that these considerations do not contradict the paradigm that Ngb is still neuroprotective in vivo by controlling O_2_ distribution in the cell, perhaps in concert with ROS detoxification, but the exact role of Ngb in the response to hypoxia deserves further investigation. However, it has been shown that Ngb mediates the hypoxia-driven neuroprotective mechanisms via HIF-1α overexpression [110] and blunts the oxidative stress in cultured mouse cortical neurons exposed to hypoxia/reoxygenation [111] and in Ngb-transgenic mice exposed to ischemia/reperfusion [112]. As it binds to excess neurotoxic NO [113] and attenuates ROS/RNS accumulation and lipid peroxidation in PC12 cells treated with H_2_O_2_ [114], Ngb may be considered a powerful anti-oxidant molecule. Ngb is also under consideration as a target for endogenous neuroprotection against stroke and neurodegenerative disorders [115]. Being a heme protein, the redox state of Ngb might have relevant effects on downstream functions. Severe in vivo hypoxia (F_I_O_2_ = 0.078 for 7 days), in facts, results into oxidation and degradation of Ngb, with marked reflections on HIF-1α and the nuclear factor (erythroid-derived 2)-like 2 (Nrf2) stabilization as well as cytochrome c release, whose outcomes include Ngb-mediated triggering of neuronal apoptosis and blunting of survival pathways [110]. 

As to our knowledge no data is available on the effect of hyperoxia on Ngb, further investigation into the neuroprotective role of this molecule in response to hypoxia and hyperoxia is still needed.

## 9. Nitric Oxide

Well-known as a master regulator of the vascular tone and blood pressure, this endothelial-derived relaxing factor improves the O_2_ delivery to cells by increasing the cerebral blood flow in the microvasculature in several pathophysiological contexts, including acute and chronic hypoxia. The formation of NO (reaction 2) is catalyzed by a family of nitric oxide synthases (NOS), which is composed by at least three isoforms, with different intracellular localizations that reflect various functions of NO.

1. nNOS (NOS1), constitutively expressed in the neuronal cells of the central and peripheral nervous systems, drives the formation of NO that acts as neurotransmitter, enhancer of neuronal synaptic activity, memory, release of other neurotransmitters and prevention of apoptosis [116].
2. iNOS (NOS2), expressed in glial cells, astrocytes, microglia and oligodendrocytes drives the formation of NO that acts in response to inflammation and cytokines.
3. eNOS (NOS3), expressed in the vascular endothelium drives the formation of NO that regulates brain circulation, and is also found in neurons and astrocytes as well [117,118].

NO plays a crucial role during chronic hypoxia. Tibetan highlanders dwelling at F_I_O_2_ = 0.12 display higher exhaled NO [119] and higher blood concentration of the NO metabolites, nitrates and nitrites [120], than sea-level dwellers. The mutation in the HIF-2α gene, critically linked to the capacity of Tibetans to adapt to high altitude [121], has been postulated to increase NOS activity and/or steady-state levels of NO signaling molecules such as nitrosothiols [122]. In addition to these key observations, several clues converge in depicting tight regulation of NOS iso-enzymes by hypoxia in the brain. The eNOS-driven signaling path plays a neuroprotective role by preserving the cerebral blood flow and supporting the vessel autoregulation process [123]. Furthermore, eNOS-driven NO protects neurons by triggering several alternative protective mechanisms that include: (a) the regulation of BDNF expression [124,125]; (b) the stabilization of HIFs [126], key to the initiation of the response to mild hypoxia [127]; (c) the *S*-nitrosylation of the HIF, PHD and pVHL proteins [128]; (d) the inhibition of the activity of PHD by interacting with the iron-binding process, thereby blocking HIF-1α degradation [129]; (e) the interaction with MAPK and PI3K signaling [130]; (f) the up-regulation of EpoR expression contributing to the Epo-mediated neuroprotection, as observed in cultured neurons [131]. When produced beyond critical levels, especially from hyperactivity of nNOS and iNOS, however, NO becomes neurotoxic because, being a free radical, damages proteins, impairing the mitochondrial function and inducing apoptosis [132].

The Km value for O_2_ of the overall NOS activity in brain, 17 mmHg [133], indicates that the NO production rate is dynamically linked to F_I_O_2_. Therefore, breathing atmospheres with F_I_O_2_ > 0.21 is expected to increase NO production due to the mass-action law. Indeed, increased NO production represents the rationale underlying hyperbaric O_2_ therapy for wound healing [134]. Furthermore, the Km value for O_2_ of the nNOS isoform (158 mmHg [135]) suggests that NO production by nNOS would increase in hyperoxia. In support of this, hippocampal and striatal dialysates of rats treated for 2 h with F_I_O_2_ = 1 at 3 atm show marked increase in the nitrite/nitrate level that is blocked by the NOS competitive inhibitor *N*_ω_-nitro-l-arginine methyl ester (l-NAME) [135]. Increased NO production in acute hyperoxia may be viewed as a protective response because it balances the O_2_-induced vasoconstriction. In fact, phosphodiesterase-5 blockers oppose normo- and hyperbaric hyperoxic vasoconstriction and accelerates seizure development, side effect of O_2_ toxicity on the central nervous system [136]. However, additional factors complicate the relationship of NO with hyperoxia. First, hyperoxia regulates the expression of NOS isoforms. In two independent studies conducted on immature rat pups, F_I_O_2_ = 0.8 for either 6 h or 6 days up-regulates iNOS mRNA in certain brain regions causing cell damage via nitrating agents overproduction [137,138]. Second, hyperoxia inactivates NO through excess release of superoxide anions suppressing the basal vasorelaxing action of NO and leading to marked vasoconstriction in the brain capillary network [139]. Another consequence of the reaction of NO with superoxide anions is the formation of peroxynitrite (ONOO^−^), a destructive radical capable of nitrating tyrosine residues in many proteins to form nitrotyrosine adducts, which therefore may become reliable markers of cell damage, because its level is inversely proportional to neuroprotection [140]. While hypoxia, or neonatal asphyxia, upregulates nitrotyrosine formation [141], the effect led by hyperoxia is not as clear. On one hand, immature rat pups exposed to F_I_O_2_ = 0.8 for 6 h display higher nitrotyrosine staining in the apical dendrites of neurons [137] (but the PO_2_-dependent proteins nitration appears to be controlled consistently by NO concentration [142]). On the other hand, human microvascular endothelial cells cultured at F_I_O_2_ = 0.95 for 72 h do not show appreciable changes in neither the nitrotyrosine levels nor the eNOS mRNA and protein expression levels, regardless of PO_2_ [143]. Observations gathered in rats exposed to 2.8 atm show that the augmented NO synthesis is associated with increased nNOS activation, Hsp90 and intracellular calcium entry [144]. Another study shows that acute hyperbaric oxygenation (5 atm) suppresses cerebral vessel vasodilation through inactivation of eNOS by superoxide anions, whereas late effects depend upon both eNOS and nNOS [145]. It should be pointed out that most of these studies concerned rather extreme situations, and inhalation of NO at low doses (5 ppm) is instead neuroprotective in rat pups exposed to hyperoxia (F_I_O_2_ = 0.8 for 8 days), because NO upregulates BDNF, thereby decreasing white matter inflammation and cell death [146]. NO was also found to counteract the hyperoxia-induced proliferation and pro-inflammatory responses of mouse astrocytes through inhibition of the activity of cycloxygenase-2 and prostaglandin E2 [147].

## 10. Carbon Monoxide

Recognized mainly as a toxic molecule because it binds to hemeproteins with an affinity greater than that of O_2_, carbon monoxide (CO) is considered deleterious for brain function [148]. But when the partial pressure of CO (pCO) is below a threshold level, CO has an important role in the activation of the carotid bodies [149] and in triggering neuroprotection [150]. More specifically, whereas free plasma CO contributes to pCO and is dangerous, the toxic effects of the same amount of CO is attenuated, if not suppressed, when CO is bound to Hb [151]. This pioneer observation reinforces the consideration of CO as a small gaseous molecule with a potential number of remarkable protective effects [152]. This vision led to the development of engineered CO-releasing molecules or CORM with anti-inflammatory, anti-apoptotic and vasodilatory mechanisms [153,154,155]. The same goal of delivering minute amounts of CO in the circulation is exploited also by infusing Hb molecules bound to CO (CO-Hb), with marked cardioprotective features [156]. 

A physiological amount of CO-Hb, which occurs in the blood of non-smoker healthy subjects, is a fundamental messenger needed for normal brain function [154]. The small increase in CO-Hb consequent to the inhalation of atmospheres containing 125–250 ppm CO protects the brain from the injury derived from 90-min transient focal ischemia followed by 48 h reperfusion [154]. Likewise, transfusion of CO-Hb is beneficial during transient focal cerebral ischemia [157]. Endogenous CO is produced in reaction [3] catalyzed by heme oxygenases (HO), a family of iso-enzymes that convert heme to biliverdin using O_2_ and NADPH as substrates. Three different isoforms of HO are expressed in brain:

1. HO-1, inducible by oxidative stress, hypoxia, heavy metals, cytokines, which is expressed constitutively only in few cerebral cell types [158,159] but nevertheless confers substantial protection via Nrf2-mediated transcriptional control of protective factors [160].
2. HO-2, expressed constitutively in brain, whose expression is much higher than that of HO-1 and HO-3, and covers nearly the entirety of the brain HO activity and CO production [161]. HO-2 expression is up-regulated in the hypoxic and ischemic brain, with increased CO production and consequent neuroprotection [162,163].
3. HO-3, a poorly known isoform that shares high homology with HO-2 at the nucleotide (88%) and protein (81%) levels, seems be a non-functional isoform as the HO-3a and HO-3b genes have the characteristics of pseudogenes derived from HO-2 transcripts [164].

The beneficial effect of CO has been associated to the overexpression of the Nrf2 transcription factor responsible for up-regulating HO-1 [150] as well as to the positive effect of Bcl-2 on mitochondrial oxidative metabolism to enhance cytoprotection against apoptosis in astrocytes [165,166]. The very high affinity for O_2_ of HO-1 and HO-2 [167] and the apparent Km value of 5 mmHg [168] suggest that O_2_ determines HO activity only at PO_2_ values < 10 mmHg, i.e., very severe hypoxia. Unlike NOS, which has a Km for O_2_ of 17 mmHg (see above), endogenous CO production by HO can thus occur in moderate hypoxia, thereby suggesting a potential role for CO as a vasodilator during tissue hypoxia, and lack of significant effects led by hyperoxia. 

At present, little data univocally support a role of CO in hyperoxia, but the competition of O_2_ with CO for the same binding site in the heme proteins indicates a potential relationship between O_2_ and CO. On one hand, this leads to the employment of hyperbaric O_2_ as elective therapy for CO poisoning [169], because the restoration of cerebral energy metabolism may become an important factor for brain activity recovery [170]. On the other hand, the inverse correlation expected between O_2_ and CO, e.g., the Warburg hypothesis, was not confirmed on an experimental ground, possibly because of a complex relationship between heme release and the stimulation of endogenous CO production by HO [171]. 

## 11. Oxidative Stress

**Figure 2:**
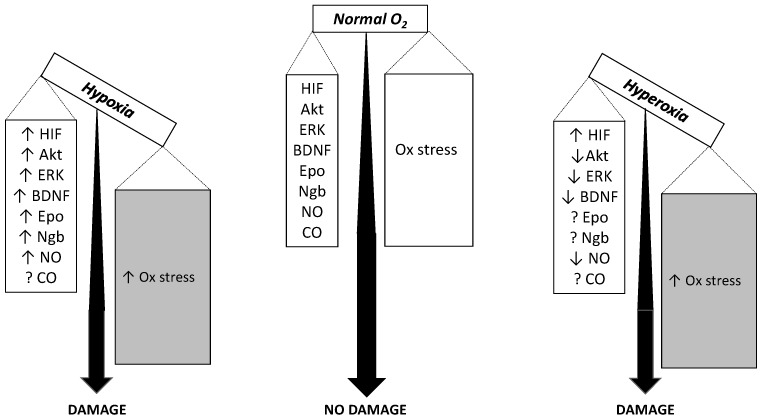
Balance between harmful oxidative stress (Ox stress, right box) and protective antioxidant defense (left box). With respect to a normoxic condition, moderate chronic hypoxia increases oxidative stress to a greater extent than moderate chronic hyperoxia, as indicated by different grey intensities. But it also increases the protective antioxidant defense to a greater extent than moderate hyperoxia (see the behavior of the various markers). The overall result is that both hypoxia and hyperoxia lead to an imbalance of the equilibrium between reactive O_2_ species (ROS) generation and the anti-ROS defense, which finally cause neuronal apoptosis and damage. Downward arrows, upward arrows and question marks mean decrease, increase and unclear effect, respectively.

An expression that describes the outcome of excessive oxidative challenge with respect to antioxidant defenses, the oxidative stress results from enzymatic or non-enzymatic transfer of a single electron to the O_2_ molecule to form the superoxide anion, which rapidly dismutes to form ROS. Although many enzyme-catalyzed biological reactions may be involved, the mitochondrial electron transport chain is considered the main site of superoxide formation as it contains several redox centers that leak electrons to O_2_, thereby constituting the major source of ROS in most tissues [55,172]. Hypoxia is known to cause ROS formation through mitochondrial uncoupling [173]. The crosstalk between mitochondria and plasma membrane NADPH oxidase [174] engages NADPH oxidase as an additional relevant source of ROS [175] and a target for several neurodegenerative disorders [176]. Neurons are highly vulnerable to the ROS deleterious effects [51,177]. Because the rate of superoxide anion formation is primarily controlled by O_2_ availability as for the mass-action law, it is expected that hyperoxia eventually leads to higher ROS formation than hypoxia just for this cause [178]. F_I_O_2_ = 0.95 for 3 h indeed stimulates superoxide anion and NO production in the caudal solitary complex of rat brain slices [179]. Early observations showed that F_I_O_2_ = 1.0 for up to 48 h causes significant pro-oxidant damage in rat brain [180]. Additionally, F_I_O_2_ = 1.0 for 60 h causes significant oxidative stress damage in guinea pig brain mitochondria with lipid peroxidation and protein modification, that was associated to insufficient increase in antioxidant superoxide dismutase activity [10]. Consistent divergence exists, however, as for the response of cells and organs to less extreme hyperoxic conditions, which led to the hypothesis that the localization of anti-oxidant systems and the activity of the various isoforms of superoxide dismutase may control differently the upregulation of pro-survival pathways [181]. In chronic conditions, the changes in antioxidant defenses are expected to become critical. Besides their toxic effects, ROS often have second messenger actions by up-regulating the expression or activating of protective genes or proteins in a feedback loop where ROS fuel the defense mechanisms against ROS themselves. However, the timing of this loop may vary considerably, leading to considerable literature controversy, and many studies failed in documenting a ROS-driven damage in short-term hyperoxia [182,183]. When moderate hypoxia (F_I_O_2_ = 0.1) and hyperoxia (F_I_O_2_ = 0.3) are applied for the same time duration in the same type of mice, they induce comparable neuronal apoptosis [9]. Although hypoxia increases ROS by a greater extent than hyperoxia, concomitantly hypoxia also increases the antioxidant defenses a greater extent than hyperoxia. The overall result is that the outcome in terms of neuronal apoptosis is comparable in the two conditions (Figure 2).

An additional important role may be attributed to glutathione, a tripeptide thiol with many physiological functions including defense and detoxification against ROS. The ratio between the oxidized (GSSG) and the reduced (GSH) form reflects the cell redox status [184]. The high vulnerability of neurons to ROS is also linked to the rather low activity of glutathione peroxidase at least with respect to other districts of the brain [185]. Both hypoxia and hyperoxia impair the defense constituted by glutathione. On one hand, F_I_O_2_ = 0.07 for 6 h reduces GSH and glutathione peroxidase activity in brain extracts [186]. On the other hand, the abnormally high GSSG/GSH ratio in weaning rat pups exposed to hyperoxia was associated with cell death [72]. Furthermore, studies performed at F_I_O_2_ = 0.95 for 5 days in weaning rats treated with a glutathione synthesis inhibitor showed a direct link between the reduced antioxidant defense of glutathione and the hyperoxia-induced pro-apoptotic changes [187].

## 12. Brain Injury

Whenever the defense mechanisms become exhausted for too severe/prolonged hypoxia/hyperoxia, or the collapse of the physiological reserve, tissue injury ensues with consequent neuronal damage. Due to their low regenerative potential, neuron apoptosis surges as a reliable marker of brain injury, with or without associated changes in HIF-1α [38]. Chronic hypoxia is well known to impair neuron apoptosis and damage, and a few underlying putative mechanisms have been worked out, including the depression of the NO/cGMP pathway with associated increase in p-ERK1/2 and p-p38 [188], morphological changes in hippocampal rat neurons and cell degeneration and death [189], p-Akt, NADPH oxidase subunit 4, and Nrf2 [9], synaptic connectivity [190], and others. In addition, in human models, brain injury can be assessed by investigating cognitive impairment. Although BDNF may represent a reliable plasma marker of brain damage [84], few human studies have been performed using this biomarker.

Brain injury in hyperoxia has not yet been as studied. F_I_O_2_ = 0.5 for 21 days reduces the expression of angiogenic vascular endothelial growth factor (VEGF) signaling and capillary density [51]. Unfortunately, brain damage data are lacking in this study, but neuroprotective EPO signaling is augmented in parallel with HIF-1α and HIF-2α, suggesting that brain microvascular density is controlled by HIF-independent mechanisms and is continuously adjusted by tissue O_2_ availability [51]. However, a study conducted in newborn piglets exposed to F_I_O_2_ = 1.0 for 1 h showed increased expression of the pro-apoptotic proteins Bax and Bad and reduced activation of anti-apoptotic proteins Bcl-2 and Bcl-xl, as mechanisms underlying the loss of anti-apoptotic defense in hyperoxia [8]. F_I_O_2_ = 0.3 for 28 days in mice increases neuronal apoptosis concomitantly with decreased protective p-Akt and EPO signaling [9]. Exposing pigs to F_I_O_2_ = 1.0 for 60 h resulted in accumulation of oxidized lipids and proteins and in stimulation of superoxide dismutase activity in brain mitochondria, suggesting that hyperoxia increases the anti-oxidant defense [10]. In rats pups exposed to F_I_O_2_ = 0.6–0.8 for 24 h, excess O_2_ is a potent trigger for apoptotic neuronal death in the developing brain, caused by oxidative stress and down-regulation of neurotrophins that provide tropic support to developing neurons, along two distinct mechanisms: (1) impairment of the antioxidant protection system (as from the increased GSSG/GSH ratio) and ROS production which leads to DNA damage, mitochondrial membranes damage and release of cytochrome C into cytoplasm; (2) gene expression changes and decrease of phosphorylation of pro-survival pAKT and pERK1/2 [72]. Ferroptosis, a newly discovered iron-dependent form of cell death that differs from apoptosis because it does not require caspases, ATP depletion, mitochondrial ROS generation, Bax/Bak or elevations in intracellular Ca^++^, may represent an important contributor to brain damage [191], but no data at present support a specific role of this process during hypoxia or hyperoxia.

To defend its function during O_2_ supply fluctuations, the brain may trigger mechanisms that increase the capillary density and cerebral blood flow through vascular remodeling processes driven by HIF-1α overexpression with concomitant activation of an array of HIF-1α downstream genes, especially the vascular endothelial growth factor (VEGF) [192]. Sustained hypoxia (28 days at F_I_O_2_ = 0.12) causes remodeling of capillary vessels by increasing their diameter and length [193], and 28-days exposure to F_I_O_2_ = 0.1 increases the vascular markers CD34 and PECAM-1 [9]. By contrast, in rats exposed to F_I_O_2_ = 0.5 for 3 weeks, the brain capillary density decreases with decreasing levels of VEGF protein and VEGF mRNA, despite increased HIF-1α and HIF-2α proteins [51]. Brief periods of hyperbaric hyperoxia induce vasoconstriction, thereby reducing the cerebral blood flow, as a consequence of reduced NO availability due to increased production of superoxide anions, which inactivate NO [194].

There is increasing evidence that O_2_ regulates the fate of the precursors of the central nervous system through activation of the neural stem cells (NSC) that reside in niches within the brain, whose proliferation and multipotency is enhanced by mild hypoxia [195]. The role of HIF-1α is controversial. While in some studies HIF-1α appears to be required [196], in others it only facilitates the signal transduction pathways that promote NSC self-renewal and inhibit differentiation and apoptosis [197]. Alternative mechanisms under study involve the canonical Wnt/β-catenin signaling, which is effective in HIF-1α knock-out models [198], the calcium-regulated Calcineurin-NFATc4, which is particularly enriched in hypoxic NSC [199], and ROS [200]. However, it should be pointed out that no studies were performed to assess the effects of higher than normal PO_2_, with the exception of one showing that relieving hypoxia in the developing cerebral cortex by ingrowth of blood vessels coincides with NSC differentiation, suggesting that vascularization in the hypoxic niches might regulate NSC differentiation by providing O_2_ [201]. 

## 13. Conclusions

Although chronic hypoxia is well known as a common feature in several environmental and pathologic situations, the reverse condition, hyperoxia, is not as well studied and still presents many controversial features, at least for the brain and the cerebral functions. For some functions, including the metabolic responses and the pathways related to protein kinase B and the brain-derived neurotrophic factor, hyperoxia acts as an antagonist of hypoxia and may even have positive effects. However, for other functions, including O_2_ sensing, the oxidative stress and apoptotic brain injury, both the O_2_ lack and the O_2_ excess appear to have synergistic harmful effects. As a matter of fact, however, the relative roles of hypoxia and hyperoxia is not yet clear for a number of functions. The most relevant clinically outcome is that hypoxia and hyperoxia appear both dangerous and challenging. Under the evolutionary point of view, and after geological eras of tremendous F_I_O_2_ fluctuations [202], it appears that Earth’s mammals are now adapted to survive in an atmosphere containing F_I_O_2_ = 0.21, and that any deviation in either direction from this value may have the potential to disrupt the pro-oxidant/anti-oxidant balance, which is very delicate in the brain.

## Abbreviations

Akt	Protein kinase B
ARNT	Aryl hydrocarbon receptor nuclear translocator
atm	Atmosphere
Bad	Bcl2 associated death promoter
Bax	Bcl2 associated X protein
BDNF	Brain-derived neurotrophic factor
cAMP-PKA	Cyclic adenosine mono phosphate-protein kinase A
CO	Carbon monoxide bound to hemoglobin
CO-Hb	Carbon monoxide
CORM	Carbon monoxide-releasing molecules
Hb	Hemoglobin
HIF	Hypoxia-inducible factor
eNOS	Endothelial isoform NO synthase or NOS3
EPAS-1	Endothelial PAS domain protein 1
Epo	Erythropoietin
EpoR	Erythropoietin receptor
ERK1/2	Extracellular signal-regulated kinases
FIH	Factor inhibiting HIF
FIO2	Inspired fraction of oxygen
GSH	Reduced glutathione
GSSG	Oxidized glutathione
HO	Heme oxygenase
HUVEC	Human umbilical vein endothelial cells
iNOS	Inducible isform of NO synthase or NOS2
JNK	c-Jun N-terminal kinase
l-NAME	*N*_ω_-nitro-l-arginine methyl ester
Km	Michaelis-Menten constant
O_2_	Oxygen
NADP^+^	Nicotinamide adenine dinucleotide phosphate (oxidized from)
NADPH	Nicotinamide adenine dinucleotide phosphate (reduced form)
MAPK	Mitogen-activated protein kinase
Ngb	Neuroglobin
NO	Nitric oxide
NOS	NO synthase
nNOS	Neuronal isoform NO synthase or NOS1
Nrf2	Nuclear factor (erythroid-derived 2)-like 2
NSC	Neural stem cells
p-Akt	Phosphorylated protein kinase B
p-ERK1/2	Phosphorylated extracellular signal-regulated kinases
pCO	Carbon monoxide partial pressure
PHD	Prolyl hydroxylase
PI3K	Phosphatidyl inositol 3-phophate kinase
PO_2_	Oxygen partial pressure
pVHL	Von Hippel-Lindau protein tumor suppressor protein
ROS	Reactive oxygen species
VEGF	Vascular endothelial growth factor
